# Hospital Performance Indicators and Their Associated Factors in Acute Child Poisoning at a Single Poison Center, Central Saudi Arabia

**DOI:** 10.1097/MD.0000000000002339

**Published:** 2015-12-31

**Authors:** Menyfah Q. Alanazi, Majed I. Al-Jeriasy, Mohammed H. Al-Assiri, Lara Y. Afesh, Fahad Alhammad, Mahmoud Salam

**Affiliations:** From the Drug Policy and Economics Center (MQA); King Abdullah International Medical Research Center (KAIMRC) (MJ, MHA, LYA, MS); Pharmaceutical Care and King Saud bin Abdulaziz University for Health Sciences (KSAU-HS) (MJ); and Pediatrics Emergency Department (FA); King Abdulaziz Medical City, Riyadh, Saudi Arabia.

## Abstract

Admission rate and length of stay (LOS) are two hospital performance indicators that affect the quality of care, patients’ satisfaction, bed turnover, and health cost expenditures. The aim of the study was to identify factors associated with higher admission rates and extended average LOS among acutely poisoned children at a single poison center, central Saudi Arabia.

This is a cross-sectional, poison and medical chart review between 2009 and 2011. Exposures were child characteristics, that is, gender, age, body mass index (BMI), health history, and Canadian 5-level triage scale. Poison incident characteristics were, that is, type, exposure route, amount, form, home remedy, and arrival time to center. Admission status and LOS were obtained from records. Chronic poisoning, plant allergies, and venomous bites were excluded. Bivariate and regression analyses were applied. Significance at *P* < 0.05.

Of the 315 eligible cases, (72%) were toddlers with equal gender distribution, (58%) had normal BMI, and (77%) were previously healthy. Poison substances were pharmaceutical drugs (63%) versus chemical products (37%). Main exposure route was oral (98%). Home remedy was observed in (21.9%), which were fluids, solutes, and/or gag-induced vomiting. Almost (52%) arrived to center >1 h. Triage levels: non-urgent cases (58%), less urgent (11%), urgent (18%), emergency (12%), resuscitative (1%). Admission rate was (20.6%) whereas av. LOS was 13 ± 22 h. After adjusting and controlling for confounders, older children (adj.OR = 1.19) and more critical triage levels (adj.OR = 1.35) were significantly associated with higher admission rates compared to younger children and less critical triage levels (adj.*P* = 0.006) and (adj.*P* = 0.042) respectively. Home remedy prior arrival was significantly associated with higher av. LOS (Beta = 9.48, *t* = 2.99), compared to those who directly visited the center, adj.*P* = 0.003.

Hospital administrators are cautioned that acutely poisoned children who received home remedies prior arrival are more likely to endure an extended LOS. This non-conventional practice is not recommended.

## INTRODUCTION

Promotion of the clinical and economic performances for any healthcare system is an ongoing challenge and demand.^1–3^ Performance indicators, such as the average length of stay (av. LOS), have been adopted as tools for quality control and improvement to achieve locally and internationally set objectives.^2,4^ The diagnosis-related group (DRG) is an internationally recognized payment system that links certain clinical diagnoses with an expected admission and LOS.^5^ A commonly reported DRG at emergency pediatric departments is substance poisoning.^6^ In 2014, 40% of the reported poison exposure cases in the USA were children (<6 years)^7^ and in Saudi Arabia 1272 poisoned children (1–15 years) were identified by the eastern regional poison center between 2011 and 2013.^8^

The admission rate and av. LOS in poisoned children depends on a number of associated factors. Age, body structure, co-morbidities as well as the nature of poison (type, amount, exposure route) are all known to influence the severity of clinical outcomes and subsequently admission rate and LOS.^9–13^ In addition, one study stated that home remedies provided to orally poisoned toddlers had put them at higher risk for the abnormal physical examination.^6^ Therefore, substance poisoning is one of the DRGs that are linked to key hospital performance indicators that are sensitive to variant child and poison incident characteristics.

Admission rates and LOS of poisoned children have been reported in a number of studies. LOS among poisoned children ranged from 1 hour to 9 days,^14,15^ with average between 1.2 ± 1.3 and 3.1 ± 4.4 days,^14–16^ and median around 1 day.^17^ On the other hand, a study stated that 46% of admitted, acutely poisoned cases were children,^16^ whereas others stated that 13.3% of poisoned children visiting emergency departments (EDs) were hospitalized.^11^ Also, it was noted that 60% to 70% of poisoned children are usually asymptomatic; often released for home observation.^18–20^

Identifying and attempting to modify variables associated with higher admission rates and extended length of stay of poisoned children will assist hospital administrators in achieving better performance indicators. Other unmodifiable variables such as gender, age category, and poison type may serve as admission predictors for care managers and bed utilization coordinators. In literature, not all associated variables were investigated and tested among poisoned children (home remedies), a nonmedical home initiated practice that still exists in some communities.^6^

The aim was to assess the admission rate and average length of stay (av. LOS) among children complaining of acute poisoning and to identify their associated factors, at a single poison center, central Saudi Arabia. This was fulfilled by: (1) obtaining the characteristics of children and poison incident, (2) assessing their admission status and LOS, (3) identifying significant factors associated with higher admission rates and extended av. LOS.

## METHODS

### Study Design

This is a retrospective cross-sectional, poison report and patient medical record review.

### Study Area/Setting

King Abdulaziz Medical City (KAMC) is a distinguished Joint Commission International (JCI) accredited tertiary health care facility established in 1983. KAMC is a certified poison center enlisted under the National Drug & Poison Information Center (NDPIC) and responds to any public or health care professional queries regarding any poison incident. KAMC is dedicated to provide health services to the military community of Saudi National Guards and their dependents. Majority of this community reside in secured well-established compounds nearby KAMC, where primary health clinics, schools, and recreational areas are allocated for them.

Within the vicinity of KAMC, a pediatric ED has an estimate of 85 beds allocated for admissions with various emergency care levels. The pediatric ED has a team of >70 emergency specialized pediatric medical staff who provide services to numerous admissions annually.^21^ On-call toxicologists or physicians with an advanced training on toxicology are readily available at all times.

### Study Subjects and Sampling Technique

By convenience, poison reports and medical charts of children complaining of acute poisoning (medication and/or chemical substance) between 2009 and 2011 were reviewed. Inclusion criteria targeted acutely poisoned children (aged 3 month to 15 years). Cases of chronic poisoning, plant allergies, venomous bites, or stings were excluded.

### Data Collection

At KAMC, poisoned children are triaged and attained for by ED physicians and licensed oncall toxicologists. As per hospital policy, a poison report form needs to be filled and signed by the medical staff for each incident. In addition, the child's health progress is recorded electronically on a system called Q-CPR, which is later archived into the hospital medical records. Study investigators incorporated their data collection forms within the hospital poison forms (between 2009 and 2011) based on an agreement with the chairman of the department. This agreement was supported by a research scientific committee, ethics committee, and chief executive office approval memorandums.

Study investigators delivered group training sessions for a team of 35 ED pediatric physicians on how to properly obtain the informed consent and gather study-related information. Two certified clinical research coordinators from King Abdullah International Medical Research Center (KAIMRC) were also assigned and trained to follow-up daily on this process and ensure that the forms are completed properly. The research coordinators used the children's identifiers to track the admission status and calculate their length of hospital stay from Q-CPR.

Noneligible patients or those with unclear contact information were dropped out. Missing variables in the forms were dealt with statistically. Validation of the collected data was done by verifying it with the records and contacting the parents (1–2 days after the incident). Phone calls after discharge were very important as questioning the anxious and stressed parents during the initial ED visit often leads to an inaccurate description of the incident details.^22,23^

### Data Collection Tool

The components of data collection tool were:

- Informed consent: name, medical record number, date/time, contact information, signatures.
- Child characteristics: age group, gender, medical/psychiatric history, body mass index (BMI) for 2 years and above, plotted on sex-specific growth charts,^24^ and classified as underweight (<5th percentile), healthy weight (5–85th percentile), overweight (86–94th percentile), and obese (≥95th percentile). Initial ED assessment was based on the Canadian 5-level triage scale: resuscitative (I), emergency (II), urgent (III), less urgent (IV), non-urgent (V).
- Poison incident characteristics: substance type, number of agents, estimated amount, form (pill, capsule, liquid, cream), exposure route, arrival time to ED (hours), home remedies provided. This section was sourced out from the reporting forms used by the Saudi Ministry of Health (MOH) and NDPIC.
- Performance indicators: children who stayed > 24 h in the ED and/or admitted to any in-hospital ward were accounted as admissions. The admission rate was calculated by dividing the number of admitted poisoned children by the total number of poisoned children multiplied by 100 during the study period. Length of hospital stay was calculated by subtracting the time of discharge from the time of ED triage assessment in hours. Timings were obtained from medical records.

### Ethical Considerations

Confidentially of the children's information was preserved by all study personnel as part of their job requirement. A record of child identifier aided in the follow-up on outcomes and validation of data collected. Signed informed consents were stapled to the data collection forms and preserved in charts. Study investigator had no influence on parents self-reporting. This study was approved by the Institutional Review Board of the Ministry of National Guard-Health Affairs (MNG-HA), Riyadh, Saudi Arabia (Protocol#: RR08/019).

### Data Management and Analysis

Data entry and analysis were performed using SPSS statistical software (Version 22; SPSS Inc, Chicago, IL). Categorical variables such as gender, BMI group, and others were presented in frequency and percentages, whereas continuous variables such as av. LOS were presented in mean (*x*), standard deviation ( ± SD), and 95% confidence interval (95% CI). Bi-variate analysis was conducted using Student's *t* test and one-way ANOVA for continuous outcomes (av. LOS), whereas chi-square was used for categorical outcomes (admission). Binomial regression and multilinear regression were constructed to identify the significant associations with admission status and av. LOS respectively, and control for all possible confounders. Adjusted odds ratio (adj. OR) and 95% confidence interval (95%CI) were obtained. Significance level set at *P* value <0.05.

## RESULTS

### Child and Poison Incident Characteristics

Eligible study participants were 315 (92%), of which the majority (72%) were toddlers (1–3 years) (Table 1). Equal gender distribution was observed (59%:41%). Almost 58% had normal weights, whereas 77% had negative medical/psychiatric history. All their fathers were full-time employees, whereas the majority of mothers were unemployed housewives (91%). The parents’ level of education varied as 22% of fathers had a university degree, whereas 70% had a certain level of school education. Mothers were slightly more educated as 31% had a university degree and 55% had school education. The average family size was 6 members, ranging between 3 and 13. Drug poisoning was observed in 63%, whereas chemical product poisoning was exhibited in 37%. The majority of children were exposed to the poison product accidentally 92%, whereas 6% were due to over dosage of their prescribed medications (mainly in infants) and 2% were suspected cases of child abuse or suicidal attempt. The main exposure route was oral 98%. In the drug poison group, the most common poison agents were antipyretics and analgesics (n = 56), whereas in the chemical group (n = 42) it was sodium hydroxide (a household product) (Table 2). Home remedies were provided to 22% of poisoned children before their visit to ED, which included forcing the child to drink plain water, lemon juice, milk, yogurt, salt/sugar solutes, and/or vomiting by manually induced gag reflex. The time between the incident and arrival to ED was >1 h in 52%. Two-thirds of cases were nonurgent, whereas 1.3% were at the resuscitative level. No fatalities were reported in this study.

**TABLE 1:**
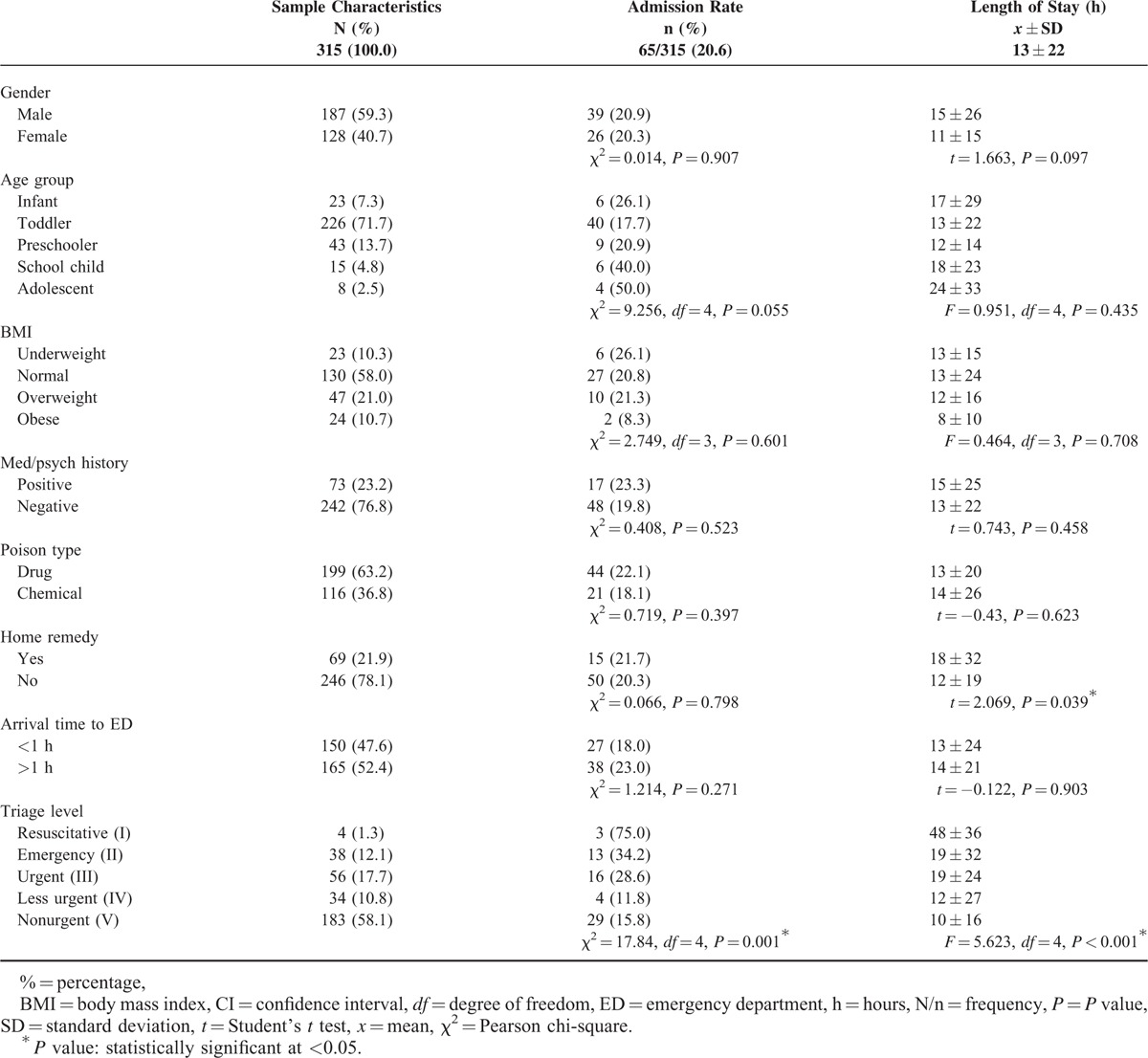
Child and Poison Incident Characteristics Compared by Admission Rate and Average Length of Stay

**TABLE 2:**
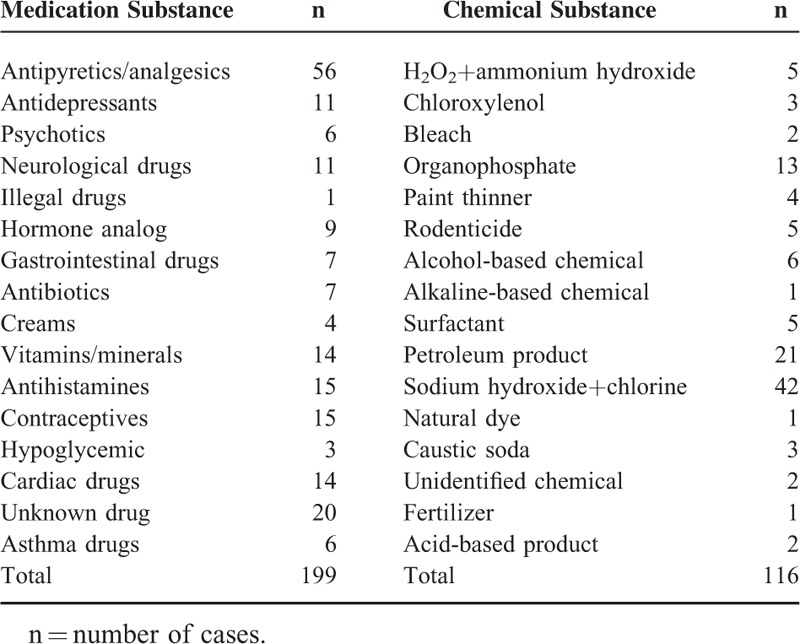
Frequency List of Poison Substances (Mutually Exclusive)

### Hospital Indicator Characteristics

Over all admission rate was 65/315 (20.6%), whereas the av. LOS was 13 ± 22 h, median was 5 h, and ranged 0.7 h to 7.4 days. Initial analysis showed that more critical triage levels was significantly associated with higher rates of admission (*P* = 0.001) and a more extended av. LOS (*P* < 0.001). Home remedies were significantly associated with an extended av. LOS (18 ± 32 h, *P* = 0.039), compared to children directly visiting the ED post incident (12 ± 19 h) (Table 1).

Binary logistic and linear regression models were constructed to further investigate the combined effect of all exposures and adjust for all possible confounders (Table 3). Older children were significantly more likely, adjOR = 1.19(1.05–1.34), to be admitted compared to younger children (adj.*P* = 0.006). More critical triage levels were more likely, adj.OR = 1.35(1.01–1.79), to be admitted (adj.*P* = 0.042). Poisoned children receiving home remedies prior ED visit had significantly endured a more extended av. LOS (Beta = 9.48, *t* = 2.99) compared to those who directly visited the ED (adj.*P* = 0.003).

**TABLE 3:**
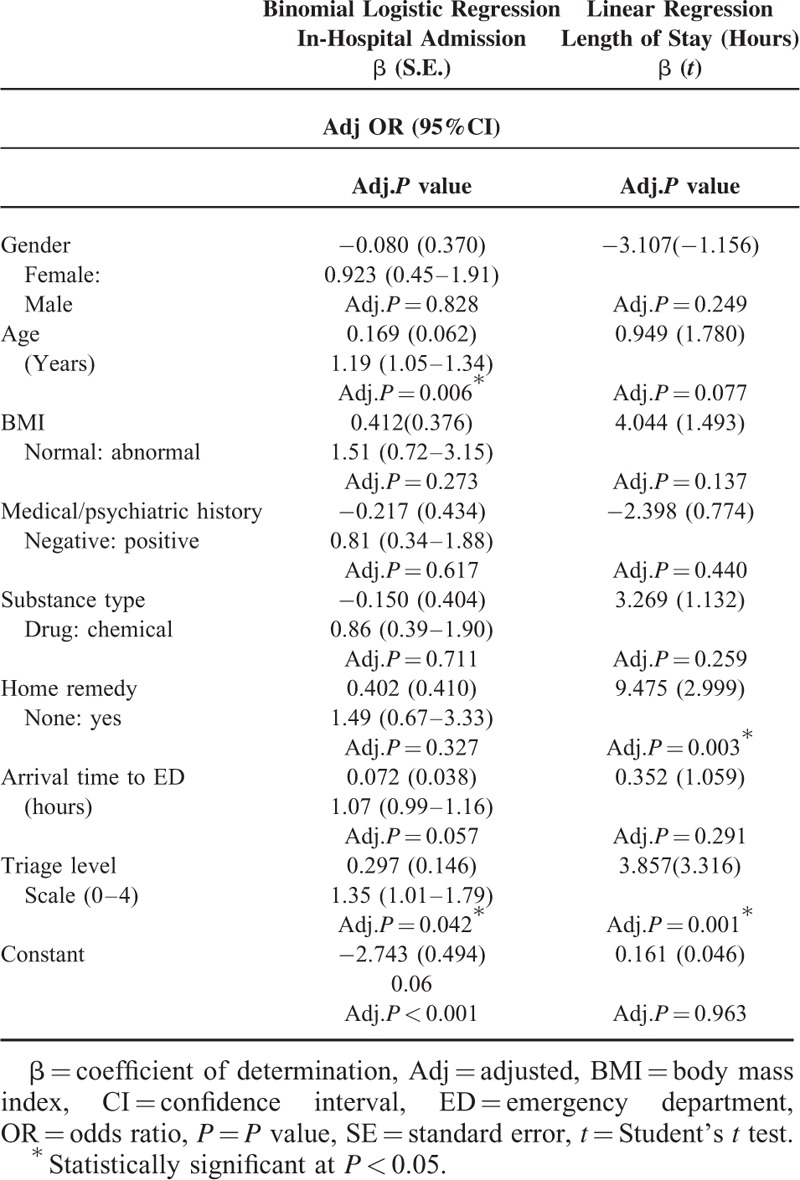
Significantly Associated Factors With Higher Admission Rates and Extended Length of Stay

## DISCUSSION

Length of hospital stay is usually affected by gender, age, arrival time to ED, mode of transport, severity of poisoning, and type of agent among poisoned victims.^25^ In this setting, poisoned children showed an overall lesser average and range of LOS when compared to other settings in USA, Finland, and an African country.^14–16^ Findings in this study indicate that the quality of performance indicators was not in favor of children receiving home remedies before their ED visit, even after adjusting for all confounders. The home remedy group has significantly endured an av. LOS (18 ± 32 h) that is a (6 ± 13 h) delay compared to the group directly visiting ED (12 ± 19 h). This rejects the null hypothesis that states such practice (as perceived by parents) improves the clinical and hospital outcomes.

Parents thought that by administering home remedies their children would not be in need for in-hospital admission, whereas in fact it slightly increased the admission rate, but with no statistical significance (20.3% vs 21.7%). More critical triage level children were found to endure higher admission rates and extended av. LOS due to the severity of clinical status. Older children seem to be more likely admitted compared to younger ages (adj.*P* = 0.006).

The type of reimbursement system or health insurance plan plays a significant role in the patient length of stay at hospitals, because reimbursable hospital services must be medically necessary, actually provided, and accurately documented in the medical records.^26^ A hospital billing department reported that the 1st hours of hospitalization for any patient admitted encompass the highest patient charges. These are the return profits that hospitals get reimbursed for the diagnostic and therapeutic procedures that patients undergo in the first hours after admission. The percentage of profit will then drop by 40% to 50% per day when a patient's LOS exceeds the average expected for a DRG.^27^ A delayed child stay at any ED congests the wards and blocks beds against potential admissions. Accelerated discharges are less expensive than standard care on all direct economic measures such as staff time and LOS.^28^ Therefore, poisoned children exceeding their predicted LOS due to such home remedies are slowing the bed turnover within the healthcare system and decreasing profits.

Patient satisfaction is another great concern that is inseparable from care, quality, and hospital operations.^29^ Studies stated that shorter length of stay showed more satisfaction and elevated stress off panicking parents after a poisoning incident.^29,30^ In this study, advising parents to abide with poison management guidelines and refrain from administering home remedies will eventually promote early discharge, minimize the chance of contracting nosocmial infections, and boost patient satisfaction.

The characteristics of child and poison incidents in this study were comparable to a number of published studies. In the USA, toddlers are indeed the highest risk group among children (73%) which is similar to the figures in this study 226/315 (72 %).^11,31^ In addition, no gender difference in poison exposures was reported by the Spanish society of pediatric emergencies,^9^ which was similar to the finding in this study. Studies in the region stated that paracetamol and other analgesics,^12,13,32^ as well as pesticides and household products^12^ were the most common ingested substances. In this study, antipyretics were indeed the most common, but the most common chemical was sodium hydroxide products followed by petroleum products. In literature, oral poison ingestion remains a leading exposure route,^13^ likewise in this study (98%). Majority of study participants upon triage were classified as non to less urgent (68%) which is comparable to the Irish 2013 poison center report stating that 70% of their cases were symptomless.^12^

Home remedies are non-medical practices that still exist in some communities despite the fact that it is not recommended by poison management guidelines.^10,33^ The 2 main types of home remedies performed by parents were orally administered fluids and/or gag triggered vomiting. Food and beverages can have a profound impact on many medications taken in therapeutic doses,^34–37^ but little is known on its desired effects to treat toxic dosages in poisoning. Gag triggered vomiting in this study was a risky and unpleasant practice that exerted physical and psychological stress on children.^38^ Moreover, some chemical substances ingested are irritating and may damage the lining of the esophagus, pharynx, and oral mucosal surface during vomiting.

### Limitations

The present study was conducted in a single poison center that serves a military community and their dependents; therefore it might limit its generalizability. Data collection was executed over a 2-year period. Further eligible cases would have been recruited to increase the statistical power of the sample, but study investigators had to abide with the approved time limit of data collection as per the agreement with the IRB and ED personnel.

The amount of poison ingested was not accounted for as a potential risk factor due to the diverse nature and forms of the substances ingested (powder, cream, fluid, pills, capsules, etc). Amounts of these diverse substances could not be quantified using a standard measuring unit and it was reported by parents in rough estimates. The fact that home remedies were self-reported practices by parents who were under stress and fear at the time of incident is another concern. Authors suspected a recall and/or a cognitive bias from parents who were reluctant to admit the details of such practice during the initial ED visit. This was overcome by phone calling the parent at a later time to revalidate the reported practice.

## CONCLUSIONS

Home remedies provided to poisoned children before their visit to this center significantly delayed the av. length of stay, one of the key performance indicators for hospitals and reimbursing bodies. A significant association was found among poisoned children of older age and higher admission rates. The severity of clinical triage assessment was also a significantly associated factor with higher admission rates and extended av. LOS.

## RECOMMENDATIONS

The av. LOS is a simple and important indicator for care management and effectiveness in child poisoning scenarios. This study measured the av. LOS as a parameter for such a DRG so that, in the future, it is benchmarked locally and/or internationally. Cases with delayed av. LOS beyond this study finding may trigger further investigations to identify and modify other associated factors. Care coordinators and other health practitioners in EDs are now able to predict and anticipate an in-hospital admission and LOS based on the child and poison incident characteristics highlighted in this study.

Parents are recommended to adhere to the local and international poison management guidelines. Community awareness campaigns aid in orienting them on the importance of notifying a nearby poison center upon any incident. A unified hotline poison control number in Saudi Arabia is essential and its placement at homes will definitely cut-off delays in seeking treatments. Environmental characteristics, such the storage of poison products and safety seals on poisons, were questioned during the phone calls. Almost 25% of chemical products were placed in unlocked cabinets, whereas 17% were placed in living rooms, bedrooms, and kitchen floors during the incident. This necessitates the importance of launching poison awareness campaigns to orient parents on safely storing and disposing potentially poisonous products. Due to the fact that such practice does exist in the community and testing it in randomized control trials is not scientifically and ethically applicable, poison centers need to inquire further on such data from parents who commit such practice. Therefore, it is advisable to incorporate it within the Saudi MOH and NDPIC reporting forms for drug over dosage or chemical poisoning, to further investigate the spread and outcomes of such practice.
